# Self-Medication of Drugs in Nursing Students from Castile and Leon (Spain)

**DOI:** 10.3390/ijerph18041498

**Published:** 2021-02-05

**Authors:** Maria Isabel Galán Andrés, Valeriana Guijo Blanco, Inés Casado Verdejo, José Antonio Iglesias Guerra, Daniel Fernández García

**Affiliations:** 1 Department of Health Sciences, University of Burgos, 09001 Burgos, Spain; migalan@ubu.es; 2 Department of Nursing and Physiotherapy, University of León, 24071 León, Spain; ines.casado@unileon.es; 3 Department of Nursing, Palencia University School, 34004 Palencia, Spain; jaiglesias@fecyl.com; 4 Intervention Radiology Nurse, Complejo Asistencial Universitario de León, 24071 León, Spain; dfernandezg@saludcastillayleon.es

**Keywords:** education, nursing students, prevalence, related factors, responsible self-medication, self-prescription

## Abstract

To determine the prevalence of self-medication in nursing students and their related factors, a transversal, descriptive study was performed on a sample of 378 nursing students. A total of 73.8% of the sample declared having used off-prescription drugs during the last month (2.84;2.26–3.58). A total of 28.9% said they did this because they are familiar with the health problem and its pharmacological solution and 25% deemed that it was a mild health problem. Drugs most commonly used off-prescription were analgesics in 88.91% (3.63;2.74–4.80) of occasions. They were mainly recommended by the students’ family (1.31;1.03–1.65) on 58.12% of the cases. Students keep analgesics they take off-prescription in their home first aid kit (4.47;3.28–6.08; *p* < 0.001). Unlike other studies, 53.2% obtained off-prescription drugs from the home first aid kit (1.13;0.89–1.43; *p* < 0.001). In addition, they gave advice and recommend drugs they have taken to other people with similar symptoms (1.97;1.59–2.44). A total of 85.72% kept excess drugs after a treatment (6.00;4.50–7.99). Self-medication is related to the storage of unused medicines and giving advice on the use of drugs to other people, among other things. Self-medication of drugs among nursing students is high. Thus, it appears necessary to review the training on rational the use of drugs and responsible self-medication in the discipline’s curriculum.

## 1. Introduction

Self-care is the will and capacity of people to take part in an intelligent, autonomous, and informed way in decisions regarding their health. Self-care activities include self-medication. Self-care is the patient’s own decision, which entails the use of drugs without healthcare professionals intervening.

When an individual decides to use a drug without medical prescription, they take their own idea or that of others as a reference about the product’s efficacy, which is related to prior experiences, reading an information source or the novelty about a drug, among other reasons.

The concept of self-medication has evolved in the last few years. Initially, it came close to self-prescription. However, the two must be differentiated because of their practical implications. Self-prescription is the self-consumption of drugs that require a prescription because of their indications or risks. However, the user forgoes intervention by a health professional [1].

Currently, this has led to the concept of self-medication being confined to the use of over-the-counter (OTC) drugs that do not require prescription, the so-called “self-care drugs”. They also include cosmetic and dermopharmacy products, dietary complements, phytotherapy, self-care and hygiene health products that could be bought based on the recommendation of a health professional.

Since the aforementioned Declaration of the World Medical Association (WMA) on self-medication [1], adopted by the 53rd General Assembly in Washington in 2002, and restated by the 191st -Council Session Prague, Czech Republic in 2012-, health authorities promote what has been dubbed responsible self-medication, a term that determines the decision of people to use drugs by themselves to treat mild conditions or specific symptoms.

This entails a person’s initiative to take part actively, autonomously, and responsibly in the management of diagnosis, prevention, and treatment in certain health–disease situations, such as the control of symptoms during mild processes. It also implies the need for drug consumption to be timely. Moreover, if the problem does not go away a professional should be sought [1]. However, self-prescription encouraged by custom, verbal, family or social advice, free of any fundamental or scientific evidence, is not responsible.

The World Health Organization (WHO) sees responsible self-medication as a valid care formula in developed societies [2,3]. The WHO has proposed rational drug use as a strategy defined as the correct and suitable use of drugs that prevent adverse effects of self-prescription [4]. However, inappropriate self-medication and self-prescription may be forms of irrational drug use.

For this topic, patient safety is also an aspect that must be recalled when tackling self-medication and self-prescription. This is because patient safety implies that “correct knowledge of care risks, removal of expendables and prevention and protection against those risks that inevitably must be assumed” [5].

In Spain, the prevailing legislation does not enable dispensing medically prescribed drugs without a prescription [6]. However, in other cases, such as over-the-counter (OTC) drugs, they can be dispensed without a prescription.

According to data from the series of the Spanish Health Survey [7] and European Health Survey [7] in regards to the consumption of non-prescribed drugs in Spain, in the last 20 years this percentage peaked in 2003 at 18.11% and subsequently fell to 13.53% in 2011. However, in 2014 the percentage rose to 18.71%, the highest on record since these surveys were introduced. Moreover, according to the Drug Observatory of the Corporate Federation of Spanish Pharmacists [8], Spain ranks fifth for drug consumption by self-medication after Italy, United Kingdom, Germany, and the United States.

Against this backdrop, the data obtained by the Centre for Sociological Research [9] reflects that 16% of Spaniards keep entire packages of drugs prescribed by a doctor because they were prescribed in advance so that the patient does not run out (52.9%); modifications to the treatment so they were not consumed (31.6%); or that the patient decided not to take them (19.5%).

Thus, self-prescription in Spain is one of the widest forms of self-treatment, and so in future health professionals, responsible self-medication has individual and social benefits that justify its analysis. However, in the last 20 years there have been few studies performed in Spain on self-medication in university students. This is not the case in South America.

Therefore, the study of this practice in university healthcare sciences students is of interest as they will be qualified professionals in the future and will have an impact on their setting. Moreover, the university context offers an opportunity to propose awareness and training actions on responsible self-prescription and self-medication.

The aim of the present study was to determine the prevalence of self-medication drugs in nursing in Castile and Leon, Spain.

## 2. Materials and Methods

This was a transversal, descriptive study performed during the academic year 2016–2017 in the region of Castile and Leon (Spain).

The population comprised 2912 undergraduate nursing students over four academic years. In a non-probabilistic sample, the necessary size was 378 students for a maximum admitted error of 4.7% and a confidence level of 95%.

In prevision of resignations or incorrect filling in of questionnaires, the invitation was increased by 10%, whereby 415 surveys were sent out among first to fourth year nursing students in during the last three months of the academic year.

The inclusion criterion was any graduate nursing student who agreed to answer the survey. Questionnaires with inconsistent answers and those with fewer than 15 of the 19 questions answered were excluded.

Before filling out the survey, students were asked for their verbal consent and notified about the voluntary nature, anonymity, and confidentiality of their answers. Moreover, they were notified about the differences between self-medication and self-prescription.

The survey was self-designed because there is no validated instrument for this population in Spain. To draw up the survey, a bibliographical review was performed about self-medication and self-prescription in university students based on the following bibliographical data: PubMed, Crochane Plus, Virtual Health Library (BVS), Spanish Medical Index (IME), National Health Sciences Library (IBECS), Latin American and Caribbean Literature in the Health Sciences (LILACS), Scielo, Scopus, Web of Science (WOS) and Cuiden Plus. This search was performed in November 2016, and the instruments used in other studies were drawn from this. The preliminary instrument that approximated to our aims was drawn up from these.

In January 2017, there was an evaluation of five experts from the Healthcare Sciences selected for their professional relevance (academic and research). They were presented with 25 items from which they had to evaluate their coherence, relevance, and clarity according to the proposal by Escobar-Pérez and Cuervo-Martínez [10]. A second preliminary instrument was drawn up from their contributions. In February 2017, a pilot study was performed with fourth year students from one of the faculties, which was later excluded from the sample. Questions from the evaluation of the instrument were added to the questionnaire related to the required time to complete it, potentially ambiguous or poorly formulated questions, subjectivity or insufficient options to answer. The questionnaire was drawn up again with suggestions from the students and it was resubmitted for them to verify.

The psychometric properties of the questionnaire were evaluated by means of reliability and exploratory factorial analysis. The reliability test for Cronbach’s internal consistency (alpha value: 0.724) was appropriate for this kind of document. The Kaiser–Meyer–Olkin (KMO) measurement of suitability of the sample attained a value of 0.497 (*p* = 0.000), which enabled an exploratory factorial analysis of principal components and Oblimin rotation with Kaiser normalization. It was detected that all items were involved in variance, albeit with variable weights (from 12,744 to 1819).

The final draft of the questionnaire was structured into three sections with nineteen questions, mainly policotomic. Four were sample classification questions; ten were related to self-medication; and five to self-medication.

The sample classification items were age, gender, course, and academic year. Regarding self-medication the questions covered the reasons for self-medication, the frequency, type of drugs consumed and its type, the people who recommended using a certain drug, the place of acquisition, and the drugs they have at home. In relation to the responsible self-medication, the questions explored which professional recommended the drug, the reading of the information included with product, what is done with excess drugs and whether these are stored at the professional’s home and why.

Together with these issues, the dependent variables were whether or not drugs were prescribed and which healthcare professional recommended a drug not subject to prescription.

Data analysis used measures of distribution, frequency, and prevalence, in regard to Kendall’s Tau, Spearman’s Rho and Spearman’s chi-squared correlations of association and effects together with the odds ratio and multivariate analysis by means of logistic regression.

The statistical significance level assumed in this study was 5% calculated for 95% confidence intervals. Statistical programs used were PSPP^R^ 1.0.1 by GNU Project and EpiDat^R^ 4.1 of the Regional Government of Galicia Regional Department of Health (Spain), the Pan American Health Organization (PAHO-WHO) and CES University of Colombia.

## 3. Results

The sample that filled in the questionnaire was composed of 409 students (98.5%). The number producing data was 378, including 80.4% women. The mean age was 20.56 years (SD 3.01; 17–42). A total of 70% of the sample were 19 or 20 years old (Table 1).

A total of 73.8% of the sample declared having used off-prescription drugs during the last month (*p* < 0.001). Conversely, 66.4% of those surveyed stated that they recommend drugs they have taken to other people with signs and symptoms similar to those they had (*p* < 0.001). (Table 2).

Of those that self-medicated in the last month, 40.1% did so routinely once a month (*p* < 0.001).

A total of 46.9% stated they do so because they are aware of the health problem and its pharmacological solution (*p* < 0.001); 25% did so because they deemed it was a health problem or mild abnormality (*p* < 0.001). However, differences were not statistically significant (Table 3).

The most common drugs used without prescriptions were analgesics, with a total of 88.91% (*p* < 0.001). The rest of the other drugs (vitamins, laxatives, antipyretics, etc.) were used with frequencies lower than 5% (*p* < 0.001) (Table 4).

Self-medicated drugs were in most of the cases recommended by the student’s family (*p* < 0.001), followed by health professionals in 58.12% and 36.6% of occasions, respectively. University friends, colleagues, and lecturers made up the remainder, *p* < 0.001.

A total of 53.2% of those surveyed stated that they obtained off-prescription drugs from the first aid kit at home (*p* < 0.001). This was followed by the community pharmacy on 43.2% of occasions (*p* < 0.001). The remaining possibilities (family members, friends, internet purchases and others) were occasional.

**Figure 1:**
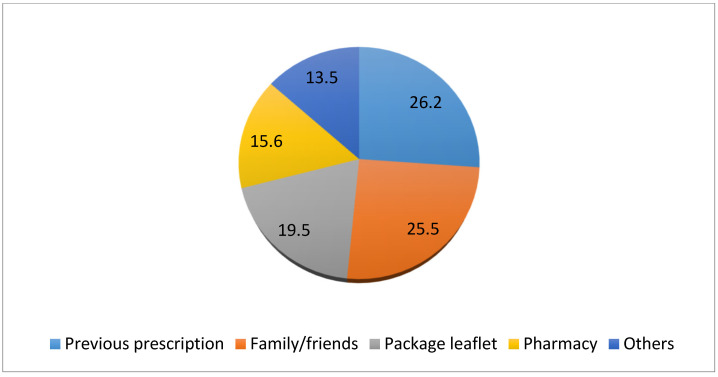
Sources of information about the product (%).

Information on the product’s indications was obtained in more than half the cases because they already knew about this due to previous prescriptions for themselves or for family and friends (*p* < 0.001) (Figure 1).

To obtain these drugs without prescription, 55.47% visited their regular or trusted pharmacy (*p* < 0.001). This was followed by any other pharmacy with 43.34% (*p* < 0.001).

Of the drugs stated by students to be part of their first aid kit and taken without prescription, analgesics were noteworthy in 81.72% of cases (*p* < 0.001), followed by antibiotics (7.86%) and anti-inflammatories and anti-pyretics, with 3.46% and 3.42%, respectively.

A total of 46.8% of the sample highlighted capsules as the dosage form most commonly used when students self-medicated (*p* > 0.001), followed by tablets and pills with 38.91%. A combination of both comprised 86.71%, and among both there were no statistically significant differences (*p* = 0.104). Sachets for dilution (8.92%) and syrups and solutions (2.96%) made up the remainder (Table 5).

A total of 61.82% of the students surveyed expressed taking prescribed drugs during time intervals of more than two months. This entailed statistically significant differences with the remaining options (*p* < 0.001). A total of 22.8% took prescribed drugs between once and twice a month, whilst 15.42% of those surveyed do so several times a month.

Regarding to the information set out in the package leaflets, regardless of whether or not they were prescribed drugs, these were consulted by students always or almost always in 64.24% of cases, whilst 22.81% never or almost never consulted package leaflets. This meant that differences were statistically significant (*p* < 0.001). The remainder expressed only doing so occasionally.

In relation to the habit of storing excess drugs after a treatment, be it self-prescribed or not, 85.72% of the sample declared doing this (*p* < 0.001). The main reason to store the drugs (66.71%) was to take them on their own account if they had the same symptoms again (*p* < 0.001). Only 8.52% left them in the Integrated Recovery System of Packaging Waste Management (SIGRE Recycling Point).

During bivariate analysis, statistically significant correlations were detected between self-medication and where information was obtained about the product (*p* = 0.046), the frequency of self-medication (*p* = 0.035), causes of self-medication (*p* = 0.030), type of preferred pharmaceutical presentation (*p* = 0.035), storage of excess product (*p* = 0.007), and giving advice to third parties on the use of drugs (*p* = 0.007). However, intensity was low in all cases (0.114 to 0.155).

No satisfactory predictive model was found based on the relative risk of self-medication in the multivariate analysis, with the logistic regression exhausted at 20 steps.

## 4. Discussion

This study responded to the twin objectives of establishing the prevalence of self-prescription and analyzing the associated factors and circumstances. The results demonstrated that almost three-quarters (73.8%) of students had self-medicated over the last 30 days, and it was a recurrent behavior that was repeated month after month with relative frequency. Self-medication behavior of students was consistent with similar studies [11,12,13,14,15,16,17,18,19,20,21,22,23]. For example, at a national level, Guillem Sáiz [17] established slightly higher figures of 88.9% of health specialty students self-medicating at Valencia University, whilst Cecilia et al. [18] reported a slightly lower figure (72.5%) for the case of pharmacy students in Murcia. Using data from nursing students, other studies showed different levels of prevalence: 76% [16], 87% [19], 88.24% [20], 92.93% [21], and 98% [21]. The studies comparing students from different health degrees—pharmacy, medicine, nursing—showed contradictory findings: for example, Johnson et al. [21] indicated that healthcare students showed the higher levels of self-medication, whilst Al Essa et al. [23] concluded the opposite.

In this study, the homogeneity of the sample from an age perspective did not allow us to assess the differences between younger and older students, because most of them were between 19 and 20 years old. The same happened with the academic year they were in, which did not maintain a significant relationship with self-medication. These factors have been analyzed in other studies and appeared as a constant: older age and higher academic years correlated significantly with higher frequency of self-medication [19,24]. Nevertheless, no differences were found in groups of pharmacy students between first- and fourth-year students [25].

Among the determining factors of self-medication was the habit of keeping excess medication following treatment, whether self-medicating or not: a behavior found among 8 out of every 10 of the students surveyed (*p* < 0.001). This aspect has received little attention in other studies, although Helal et al. [26], pointed out that 77.5% of university students in Egypt had medicines stored at their first aid cabinet, and this was significantly associated with self-medication. Thus, keeping medicines at home is an intentional behavior anticipating future health problems, either for similar or other ailments. If they do not have a first aid cabinet at home, students expressed to buy them from a pharmacy, with a preference to buy from a pharmacy they already knew (*p* = 0.001). These findings were higher than those found in other studies, such as García Ávila et al. [22] with a Colombian population and Ávila Baeza et al. 2017 [27] in a study conducted in Mexico. González-Muñoz [28], using a sample from the University of Cordoba (Spain), showed similar results. However, to evaluate the variability it is important to consider the differences between countries in the accessibility to pharmacies, and the legislation could restrict the access to medicines without prescription, because they differ greatly from one country to another.

The drugs most commonly used without a prescription were analgesics (88.91%), followed by antibiotics (7.86%), anti-inflammatory medicines (3.46%) and antipyretics (3.42%). Similar figures were obtained in most of the studies that were consulted [19,29,30,31,32]. Although the works of Kumar et al. [33] and Johnson et al. [21] showed a greater usage of antipyretics, it is important to note that in many antipyretics, the active ingredients are analgesics. Patil et al. [12] established that antibiotics were most commonly self-medicated (63.91%) by students. The type of drugs most used when self-medicating is consistent with the most common health issues encountered in young people, which usually entail mild symptoms.

From the perspective of the type or format of the medicaments, the most frequently self-medicated medicines by the students were capsules (46.8%), followed by pills and tablets (38.91%), sachets (8.92%), and syrups and solutions (2.96%). In total, it meant that 97.59% prefer an oral medicine, which was consistent with the findings of Ávila Baeza et al. [27].

In our study, 46.9% of the students indicated their knowledge of health problems and their pharmacological solution as the main reason for self-medication, in contrast with other studies [21,22,29] which showed higher percentages of students self-medicating because of their knowledge, ranging from 36 to 42%. Our finding is consistent with the work of Goel et al. [20], who found that only 22.86% of nursing students surveyed self-medicated as a result of their learning opportunities. Therefore, it seems that other factors such as the severity of the diseases, recommendations received from family members, or having prior information of the product influenced the decision to self-medicate. Even if the students did not have the knowledge, almost 66.5% of the respondents had recommended medicines to others, based on their own experience and the similarity of the symptoms; thus, taking on a responsibility that goes beyond their professional role and promoting self-medication in other people.

In contrast to the inadequate habits to self-medicate, the majority of the students reported reading the information leaflet of the medications. In fact, the results indicated that once they had the medicines, regardless of whether they were prescribed or not, the instructions in the prospectus were consulted in all or most cases in 64.24% of the occasions. Only 22.81% stated to never or rarely referring to the information leaflet, which implies statistically significant differences (*p* < 0.001). Okyay and Erdogan [15] obtained similar results.

It was noted that self-medication and the related logic was preferable, independent, and individualized. It was perhaps influenced by the habits acquired at home and by the feeling of safety that might come from studying this branch of knowledge in their university studies as future nurses.

Similarities and differences have been established with other studies, but it is not easy to establish a profile of the students who self-medicate the most. As seen in the results section, a bivariate analysis has established statistically significant correlations, but in all cases with small intensities, between the frequency of self-medication and the variables—reasons, type of pharmaceutical presentation, storage of leftover product, giving advice on the use of drugs to third parties, and the place where the information on the product is obtained. Multivariate analysis with logistic regression did not offer a satisfactory predictive model based on the relative risk of self-medicating.

Some limitations may be addressed when comparing these results with other studies due to the differences between the assumed concepts of self-prescription and self-medication, reference time intervals and accessibility factors, sales points, and the possibility of acquiring drugs without prescription.

In order to determine whether self-medication is a specific problem of nursing students, the study should be extended to students from other degrees and even to the general population, because it seems that the habit of self-medication is transmitted within circles of family and friends.

## 5. Conclusions and Recommendations

Self-medication of drugs among nursing students is high. This is usually because of mild health problems. Unlike other countries, family members of students are those who most often recommend off prescription drugs, and in turn, students recommend these to third parties. Health professionals are a secondary source of information. Moreover, excess drugs are kept in their homes and comprise one of the most important sources of medicines supply.

Knowledge is the basis to generate attitudes, and this leads to health-related behaviors and lifestyles. Therefore, as future health professionals, they should be better trained on the rational use of drugs, responsible self-medication, and the essential role played by health professionals as sources of information in regard to use of drugs, whereby the discipline’s curriculum could be reviewed.

Further studies are needed on the self-medication of drugs in Spain, which, if designed homogeneously, would provide a reliable picture of the problem to be able to intervene.

## Figures and Tables

**Table 1:** Sociodemographic variables of nursing students.

Factor	***n***	Range	Min.	Max.	Mean	SD
Age	378	25	17	42	20.56	3.016
	***n***	Women	**%**	Mean	**%**	
Gender	377	303	80.40	73	19.36	

**Table 2:** Frequency of off-prescription drug use in the last month and advice on self-medication.

Factor		Frequency	Valid Percentage	*p*-Value
Off prescription drug use	NO	99	26.2	
	YES	279	73.8	<0.001
Advice on self-medication	NO	125	33.1	
	YES	251	66.4	<0.001
Total		378	100	

**Table 3:** Factors linked to self-medication.

Factor	Frequency *n* = 376	Valid Percentage
Knowledge about the problem	177	46.9
Mild health problem	94	25
I had it at home	32	8.4
I had used it before	30	8.1
Lack of time to go to the doctor	25	6.6
Other	18	5

**Table 4:** Drugs used for self-medication.

Factor	Frequency *n* = 376 (512 Answers)	Valid Percentage
Analgesics	455	88.9
Vitamin and mineral complexes	26	5
Laxatives	25	4.8
Antibiotics	24	4.6
Antipyretics	21	4.1
Anxiolytics/tranquilizers	19	3.7
Contraceptives	17	3.3
Antidepressants	14	2.7
Others	68	13.2

**Table 5:** Forms of presentation of drugs.

Factor	Frequency *n* = 376	Valid Percentage
Capsules	176	46.8
Tablets	89	23.7
Pills	57	15.2
Sachets for dilution	33	8.9
Syrups	7	1.8
Solutions	4	1.1
Other	9	2.3

## Data Availability

Not applicable.
